# Obesity as a Major Risk Factor for Cancer

**DOI:** 10.1155/2013/291546

**Published:** 2013-08-29

**Authors:** Giovanni De Pergola, Franco Silvestris

**Affiliations:** Department of Biomedical Sciences and Human Oncology, Section of Internal Medicine and Oncology, University of Bari “Aldo Moro”, School of Medicine, Policlinico, Piazza Giulio Cesare 11, 70124 Bari, Italy

## Abstract

The number of cancer cases caused by being obese is estimated to be 20% with the increased risk of malignancies being influenced by diet, weight change, and body fat distribution together with physical activity. Reports from the International Agency for Research into Cancer and the World Cancer Research Fund (WCRF) have shown that the strongest evidence exists for an association of obesity with the following cancer types: endometrial, esophageal adenocarcinoma, colorectal, postmenopausal breast, prostate, and renal, whereas the less common malignancies are leukemia, non-Hodgkin's lymphoma, multiple myeloma, malignant melanoma, and thyroid tumours. To be able to develop novel methods in prevention and treatment, we first must understand the underlying processes which link cancer to obesity. Four main systems have been identified as potential producers of cancer in obesity: insulin, insulin-like growth factor-I, sex steroids, and adipokines. Various novel candidate mechanisms have been proposed: chronic inflammation, oxidative stress, crosstalk between tumour cells and surrounding adipocytes, migrating adipose stromal cells, obesity-induced hypoxia, shared genetic susceptibility, and the functional defeat of immune function. Herein, we review the major pathogenic links between obesity and susceptibility to cancer.

## 1. Introduction

Obesity is a serious problem which heightens the risk of several chronic illnesses including cancer development [1–3]. Current recommendations from the Public Health Goals of the World Cancer Research Fund (WCRF) suggest that the median adult BMI should be maintained between 21 and 23 kg/m^2^, depending on the normal range for different populations [4]. 

## 2. Obesity and Cancer Risk

### 2.1. Epidemiology and General Factors

It has been estimated that about 20% of all cancers are caused by excess weight [5], and the Million Women Study, the largest study of its kind on women, has shown that approximately half can be attributed to obesity in postmenopausal women [6]. There are many prospective epidemiological studies which have demonstrated a direct association between overweight and cancer, even though obesity alone does not apparently heighten cancer risk in all tissues by the same amount [1–7]. 

A recent systematic review and meta-analysis of prospective observational studies [3], with 282,000 incident cancer cases and a follow-up greater than 133 million person-years, has demonstrated that the obesity and cancer association is sex specific over a wide range of malignancies, and this remains mostly true for different geographic populations. However, cancer risk in obesity is different between ethnic groups [3] in that African Americans appear rather susceptible to cancer, in contrast to Hispanics who appear to be relatively protected while the association of increased BMI with breast cancer is particularly strong in Asia-Pacific populations [3].

The International Agency for Research into Cancer (IARC) [7] and the World Cancer Research Fund (WRCF) reports [4] showed that common cancers in obese people are predominantly endometrial, esophageal adenocarcinoma, colorectal, postmenopausal breast, prostate, and renal. Less common malignancies associated with obesity are malignant melanoma, thyroid cancers [8], and leukemia, non-Hodgkin's lymphoma, and multiple myeloma [9]. 

The role that obesity plays in carcinogenesis has been brought to the fore by data such as the rapid rise of oesophagus adenocarcinoma over the past 20 years. In fact, whether oesophageal reflux is associated with adenocarcinoma [10], or whether it is favoured by obesity [11], the change in oesophageal cancer morphology from squamous to adenocarcinoma has followed the worldwide rise in obesity. A further example is provided by weight accumulation with age which is also linked to an increase in postmenopausal breast cancer risk in women who do not follow a menopausal hormone therapy regime [12], while cohort studies have shown that breast cancer risk was lowered by 50% in women who intentionally underwent weight loss higher than 10 kg after menopause [13]. In addition, the Swedish Obese Subjects (SOS) study, a large prospective study which established that bariatric surgery achieves an average of 20 kg weight reduction in obese patients with BMI higher than 40 kg/m^2^ and that matched the surgery group with untreated morbidly obese women, reported a significant reduction in cancer incidence in association with substantial weight loss on a follow-up longer than 10 years [14]. Concerning the role of childhood obesity in adult cancer, a study performed in a cohort of 2,347 subjects retrospectively evaluated cancer risk related to age and sex-specific BMI standard deviation scores (SDS) of subjects aged 2–14 [15]. Among these subjects, the risk of cancer in adult life was increased by 9% per SD increase in childhood BMI [15]. Also, an excess of weight in teenagers was linked to doubling the mortality risk of colon cancer in adulthood [16]. Because of the time lag in cancer development in the presence of adiposity, the typical follow-up is longer than 10 years in cohorts assessing cancer risk, and it is thought that this is an “average” lag period for obesity-related cancer development [17]. However, the lag period may be shorter when hyperoestrogenemia is a predominant mechanism, as it occurs in postmenopausal breast and endometrial cancer.

### 2.2. Relation with Body Fat Distribution

Apart from BMI, other anthropometric measurements, such as waist-to-hip ratio (WHR) or waist circumference (WC), are currently being investigated as indicators of adiposity and cancer risk. It has been shown that cancers that are associated more with abdominal adiposity than with BMI predominantly include colon, premenopausal breast, endometrium, and oesophageal adenocarcinoma, together with pancreas tumours [18–23]. A recent longitudinal study (median of 5.0 years), performed in participants from the Framingham Heart Study, has shown that visceral adiposity, measured by using multidetector computed tomography, is associated with cancer after adjustment for clinical risk factors and generalized adiposity [24]. As for mortality, a longitudinal study in US women has shown that WC and WHR are strongly and positively associated with cancer mortality, independently of BMI [25].

### 2.3. Effect of Weight Change

A Canadian case-control study has found that those men whose weight increased by more than 21 kg after reaching adulthood had a colorectal cancer risk 60% higher than those with a weight increase of 1–5 kg, and the association was even stronger after excluding those patients with rectal tumours [26]. Endometrial cancer has also seemingly been shown to be directly correlated with weight gain during adulthood among Japanese women [27]. Additionally, diet-induced weight loss reduces colorectal inflammation and cancer-related gene pathways in obese individuals [28]. 

## 3. Obesity and Cancer Mortality

The American Cancer Society has furnished data which suggest that overweight and obesity are correlated to liver and pancreatic cancers death rates, as are both myeloma and non-Hodgkin's lymphoma [1]. 

Data published over the last 25 years emphasize that obesity is a cause of about 20% of deaths from cancer in women while the rate is around 14% in men. These rates are second only to smoking for the number of avoidable cancers [1]. However, these data are possibly underestimated, since average weight has continued to rise in the worldwide population over the same time. Recently, Jaggers et al. found a positive multivariable-adjusted association between cancer mortality and abdominal obesity that increases the risk of cancer mortality up to 24% [29]. Epidemiological studies have also shown that obesity is a contributing factor in both increased incidence and mortality from cancers of the colon, the endometrium, the kidneys, and the breast (in postmenopausal women) [5, 6]. In the European Union, it has been calculated that not becoming overweight could reduce the annual incidence of colon cancer by up to 21,000 cases and breast cancer by up to 13,000 cases. The higher cancer death rate in the obese also accounts for a decreased survival rate, that may be due to the enhancing effects of obesity on cancer potency and progression. A study on invasive ductal breast cancer in 1,177 women found that those in the highest quartile of BMI developed tumours with a higher histological grade, mitotic cell count, and larger tumour size compared to women in the lower quartiles [30]. Furthermore, by considering solely large tumours up to 2 cm of diameter, those in the group of the highest BMI quartile largely expressed markers of high proliferation, supporting their fast growth, as compared to those with similar-size tumours in nonobese women [30]. However, it has been reported that chemotherapy dosages are often not adequate in women of excess weight receiving breast cancer treatments when compared to dosages in lean women [31], thus implying that an overall full weight-based adjusted dose would allow better outcomes.

## 4. BMI-Cancer Associations and Histological Subtypes

Several data suggest that the BMI-cancer association is typical of a number of histological subtypes within a site specific cancer. These examples include the following.

- A dose-response meta-analysis (9 cohorts: 22 case-control studies) showed that the BMI-breast cancer association is stronger for oestrogen receptor positive/progesterone receptor-positive (ER+PR+) tumours (33% increase per 5 kg/m^2^ increment for postmenopausal breast cancer), while there were no significant BMI-cancer associations for ER−PR- and ER+PR-tumours.
- Detailed pathological examination of a Swedish population-based prospective cohort (9,685 postmenopausal women not using hormone therapy) showed that the highest quartiles of BMI were associated with ductal type tumours, grade II, low Ki67 index, HER2 negativity, low-cyclin D1 expression, ERa, and PR immune-positivities, but not ERb tumours.
- For endometrial cancer, obesity is predominantly a risk factor for type I endometrioid tumours (accounting for 70% of endometrial cancers) rather than type II.
- BMI associations are stronger for the papillary subtype of thyroid carcinoma.
- Positive associations exist between BMI and cardias gastric adenocarcinomas, but not noncardia gastric malignancies.

The biological mechanisms responsible for stronger association of BMI with some cancer subtypes are not known, but these information might have implications in determining the patient's risk and the subsequent recommendations. 

## 5. Biological Mechanisms Linking Abdominal Obesity to Cancer Susceptibility

In the obese, the mechanisms which may foster or promote cancer occurrence or progression are usually classified as being of two types. They may be universal and direct in nature in that they apply to all or the majority of tumours because they have to do with the hormonal and/or metabolic abnormalities prevalent in obesity. Alternatively, they may be site specific, in which conditions can foster a particular tumour in a particular site because they have to do with the consequential effects of obesity, so leading to complications in specific tissues or organs. There is, for example, a heightened risk of cancer of the gallbladder when gallstones are present and of oesophageal adenocarcinoma as a putative effect of aggravated gastrooesophageal reflux. Also liver cancer can be a consequence of nonalcoholic steatohepatitis [32]. Certainly, various syntheses of mechanisms can operate differently in different people.

### 5.1. Diet

Both epidemiological studies in people and experiments in animals suggest that alterations in caloric intake or in quality of diet may significantly influence the risk of cancer development and progression. However, the separate effects of diet per se, as well as of positive energy balance, reduced physical activity, and increased adiposity, are not easy to determine. It is well known that caloric excess will lead to increased cancer incidence and that positive energy balance appears to promote cancer cell proliferation and tumour progression. By the same token, limited long-term calorie intake has led to a decrease in cancer incidence and extended longevity in rodents [33]. There are many studies, not withstanding the symptomatic underreporting of surveys, which document the health hazards related to diets typical of the first world, rich in calories, overabundant in alcohol and animal fats, and/or lacking in plant products [34]. By contrast, a defined association between low-cancer risk and a higher intake of fruit and vegetables has been proven. High-fibre cereal consumption has also been linked to a reduced risk of colorectal cancer, although large-scale studies do not demonstrate such a benefit [34]. The negative energy balance may be an important factor in lowering the susceptibility to cancer because of direct or indirect effects on levels of insulin [35], insulin-like growth factor-I (IGF-I) [35], and inflammatory biomarkers [36]. Interestingly, preliminary data demonstrate that a low carbohydrate insulin-inhibiting diet, associated with ketosis, is safe and correlated with stable disease or partial remission in patients with advanced cancer [37]. It is noteworthy that obesity is correlated with low-serum levels of 25- hydroxyvitamin D (25 (OH)D), confirmed by a study showing that low vitamin D levels may be responsible for 20% of the cancer risk linked to increased BMI [38]. 

### 5.2. Physical Activity

For the prevention of the onset of chronic diseases, including cancer, guidelines suggest exercise as part of the daily routine, a brisk walk or walking to work instead of driving, and/or practicing more physical activity at home, such as using stairs or gardening [39]. There have been several studies which show that physical exercise can lower breast cancer risk and that there are benefits both physical and mental for cancer patients [39]. It has also been correlated to a decrease in the risk of both colon and breast cancer development, and probably with a lower risk of endometrial cancer [39]. 

Regarding possible mechanisms responsible for cancer prevention, regular physical activity helps maintain a healthy body weight, regulates sex hormones, insulin, and prostaglandins and has various beneficial effects on the immune system [40]. The specific dose of exercise needed to optimize primary cancer prevention remains to be elucidated.

### 5.3. Insulin Resistance

The most popular hypothesis explaining the association between obesity and cancer is that of lower insulin sensitivity. Excess body weight and adiposity are directly correlated with insulin resistance, compensated for by the stimulation of pancreatic insulin secretion, and this commonly results in hyperinsulinemia. Elevated insulin serum levels favour faster growth and increased aggressiveness of colorectal [41, 42], pancreatic [41], liver [41], postmenopausal breast [41, 43, 44], and endometrial [41, 45] cancers. C-peptide is often used, preferably to insulin, as a serum biomarker of pancreatic *β*-cell secretory activity because of its longer half-life due to the lack of hepatic extraction, its lower metabolic clearance rate, and its lack of cross-reactivity with insulin antibodies. Several epidemiological cohort studies have indicated a direct correlation between circulating C-peptide levels and colorectal [46, 47], pancreatic [36, 46], liver [46] endometrial [46, 48], and postmenopausal breast [49] cancers. However, prospective cohort studies have not demonstrated any association between prostate cancer risk and elevated C-peptide levels or high insulin levels, although a more elevated risk of prostate cancer mortality for men with the highest C-peptide levels has been recently reported after adjustment for BMI [50]. Although insulin itself does not induce somatic cell mutations, it has anabolic and antiapoptotic effects, and it is thought that only supraphysiological concentrations of insulin have mitogenic effects. Interestingly, cancer cells commonly have the ability to respond to the activation effect of insulin on intracellular transduction pathways, particularly when insulin is highly concentrated, as occurs in obesity. Signalling by the insulin pathway is highly relevant in cancer development as both extracellular-signal-regulated kinase (ERK) and phosphatidylinositol-3 kinase (PI-3 K) pathways are triggered by activation of the insulin receptor (IR). The insulin receptor is highly expressed in adipose, muscle, and kidney tissues, while it is overexpressed in breast [51] and prostate [52] cancer cells as well as in most normal and neoplastic haematopoietic cells [51]. It has been hypothesized that the IGF-I receptors are the mediators for the main proliferative effects of insulin as the insulin receptor (IR) and the receptor for IGF-I (IGF-IR) show more than 50% of overall sequence homology and even 84% of homology in the tyrosine kinase domain of the *α*-subunit [43] while their ligands, insulin, and IGF-I share 40–50% homology. Because of this, insulin and IGF-I can interact either with IR or with IGF-IR [45], and both of these receptors have been associated with tumour development.

 Regarding increased tumour invasion, biochemical and functional analyses have demonstrated that ligand-induced activation of the IGF-IR kinase results in the loss of epithelial integrity, at the same time promoting cell migration [53]. In addition to the specific receptors IR and IGF-IR, coexpressed in the same cell, a third receptor, a hybrid form (IR-IGF-IR) between IR and IGF-IR, can mediate the effects of both insulin and IGF-I as a result of their high homology [46]. Interestingly, over-expression of both IR and IGF-IR in a variety of cancer tissues leads to heterodimerization and the formation of this hybrid receptor in amounts that even exceed IGF-IR content, especially in malignant breast and thyroid tissues [51]. The hybrid receptor in malignant tissues predominantly includes IGF-IR and IR isoform A, which has a higher affinity for binding IGF-I than insulin does and stimulates cell proliferation. 

### 5.4. IGFs and IGF Binding Proteins (and Their Interrelationship with Insulin and Insulin Resistance)

The indirect effects of hyperinsulinemia on carcinogenesis are attributable to the action of insulin on circulating endogenous growth factors together with their binding proteins. In hyperinsulinemia, the growth hormone receptor (GHR) is upregulated by the increase of insulin concentrations in portal circulation thus increasing GHR signalling and hepatic IGF-I synthesis [54]. Moreover, insulin decreases the hepatic expression of binding proteins of IGF-I, such as insulin-like growth factor binding proteins-1 and -2 (IGFBP-1, IGFBP-2) [55], thus leading to higher plasma levels and bioavailability of free IGF-I [2, 56]. In this context, IGFBP-1 inhibits the growth of breast cancer cells in mice with MCF-7 breast cancer xenografts, as well as hepatocellular cancer growth in mice over-expressing IGF-I and IGF-II [54]. IGF-I also exerts proangiogenic effects and induces tumour-related lymphangiogenesis. In fact, IGF-I induces the synthesis of hypoxia-inducible factor-1a (HIF-1a), which together with vascular endothelial growth factor (VEGF) [56] leads to neovascularization and metastases, as shown in a colon cancer model [57]. Moreover, through the inhibition of p53, IGF-I prevents apoptosis [56] and induces metastatic tumour spread, linked to integrins movement to the borders of migrating cells [58]. In addition to insulin and IGF-I, over-expression of IGF-II, a hormone produced in the liver, is also associated with tumour development. IGF-II signalling takes place through IR (specifically the mitogenic IR-A subtype) and IGF-IR. Another factor influencing IGF-I free levels is insulin-like growth factor binding protein-3 (IGFBP-3), which is the most profuse of the IGFBPs. Worthy of note is that visceral fat accumulation leads to lower IGFBP-3 plasma levels [59], and lower IGFBP-3 concentrations lead to higher free IGF-I levels. Moreover, IGFBP-3 exerts direct protective effects against cancer development, by inducing apoptosis through both p53-dependent mechanisms and Bcl-2 family proteins [54]. IGFBP-3 also interacts with the nuclear retinoid X receptor (RXR)-a, altering its binding to the transcription factor Nur 77 and activating the apoptosis cascade [54]. 

### 5.5. Sex Hormones

In women, BMI has been regularly correlated with breast, endometrium, and other cancers linked to hormones, since an increase in circulating sex steroids from weight excess is a well-known mechanism implicated in cancer development. Data from epidemiological studies demonstrate that the postmenopausal risk of breast cancer increases in women with heightened levels of circulating sex steroids, including dehydroepiandrosterone (DHEA), its sulfate (DHEAS), Δ4-androstenedione, testosterone, estrone, and total estradiol, and low levels of sex hormone binding globulin (SHBG). Endogenous sex hormones are active in tumour cell growth and directly mediate the effect of obesity on cancer, in particular in tumours of the breast [60–62] and the endometrium [63]. There is evidence that estrogens are mitogenic [64], regulate the expression of insulin receptor substrate-1 (IRS-1) in the breast [65], and induce free radical-mediated DNA damage, genetic instability, and gene mutations in cells. The Million Women Study [6] and previous studies have shown that in premenopausal women breast cancer risk decreases with increasing BMI, while it increases with BMI in postmenopausal women who do not practice hormone replacement therapy, possibly owing to heightened levels female sex hormones in circulation, caused by increased estradiol creation from androgenic precursors in adipose tissue through an increase in activity of the aromatase enzyme. In endometrial cancer, heightened concentrations of plasma estradiol and estrone are correlated with increased cancer risk in postmenopausal obese women [66] and elevations of estradiol increase endometrial cell proliferation while inhibiting apoptosis and at the same time encouraging IGF-I synthesis in endometrial tissue [2]. At variance with postmenopausal women, in premenopausal women a deficiency of progesterone is likely to have more influence than any excess of estrogen. As far the role of androgens is concerned, anthropometric parameters (BMI and waist circumference) and testosterone levels are inversely correlated in men [67] and directly associated in women [62]. 

The EPIC and other epidemiological studies have demonstrated that heightened plasma levels of androgens are associated with a more elevated risk of breast cancer in women both pre- and postmenopausal, therefore suggesting that androgens may also be considered as links between obesity and breast cancer [60–62]. Elevated plasma androstenedione and testosterone levels may also correlate with endometrial cancer risk in both pre- and postmenopausal women [65].

### 5.6. Adipose Tissue and Adipokine Production

Much metabolic and endocrine activities take place in adipose tissue. In obesity, there is oversecretion of deleterious proinflammatory molecules like interleukin-6 (IL-6), tumour necrosis factor-*α* (TNF-*α*), leptin, resistin, retinol binding protein-4, plasminogen activator inhibitor-1 (PAI-1), hepatic growth factor (HGF), and so forth and hyposecretion of beneficial adipokines such as adiponectin and visfatin [68]. These adipokines can be secreted from both adipocytes and stromal-vascular cells, and most of these substances are actually from macrophages and other immune cells (T-cells, etc.) that infiltrate adipose tissues in obese patients [69]. Interestingly, human adipose tissue macrophages resemble human tumor-associated macrophages and increase the expression of FAS in cancer cells, thus playing a critical role in cancer cell survival [70]. 

Adiponectin and leptin have been subject to much study in cancer development as they are the most abundant adipokines. Higher leptin levels can be a factor for fostering cancer development and progression since it is now known to be mitogenic, proinflammatory, antiapoptotic, and proangiogenic [71]. Most experimental studies on leptin have been on breast cancer, and they have shown that: (1) leptin upregulates oestrogen/ER signalling; (2) leptin intensifies the expression of aromatase and, therefore, oestrogen production [72]; and (3) leptin receptors are located in both normal and malignant breast epithelial cells. However, while breast tumour cells proliferate after leptin stimulation [73], transgenic mice with no leptin or its receptor have been shown to be resistant to tumour formation [74]. The idea has been put forward that leptin regulates hepatocellular carcinoma development through the modulation of human telomerase reverse transcriptase [75]. There are four splice variants of the leptin receptor; however, only LRb has a long enough intracellular domain to allow full signal-transducing capabilities. LRb activates the PI3 kinase, MAPK (mitogen-activated protein kinase), and STAT (signal transducer and activator transcription) signalling pathways, which are vital for cell survival, proliferation and differentiation [76], and c-fos gene transcription. Interestingly, isoforms may also have differential roles in ER-positive and ER-negative breast cancer cells [71]. 

Blocking cancer-stromal cell communication may well be a strategy to target breast cancer therapy, and, interestingly, it has been shown that leptin drives tumour progression by a bidirectional crosstalk between breast cancer cells and cancer-associated fibroblasts [77]. 

Adiponectin, being the most abundant adipokine, is particularly interesting because it enhances insulin sensitivity, and its circulating levels are inversely related to cancer occurrence and cancer stage [78]. However, adiponectin serum levels correlate negatively with BMI and even more negatively with visceral adiposity [78] while insulin and estrogens may suppress its secretion [79]. Adiponectin has a protective role in carcinogenesis, indirectly by acting against the development of insulin resistance [43] and directly, as in vitro and in vivo studies have shown, by activating the AMPK pathway [43] and inactivating MAPK kinases 1 and 3 and ERK1 and ERK2 [80]. Moreover, adiponectin stimulates apoptosis through the induction of p53 and Bax expression and the decrease of Bcl-2 expression [80], inhibits angiogenesis and cell migration [81], exerts an anti-inflammatory role, and induces downregulation of vascular adhesion molecules, thus reducing tumour spread [82]. Last, adiponectin prevents the interaction of various growth factors with their receptors [83]. Adiponectin levels have been studied in relation to the risk of several cancer types. In breast cancer, two case-control studies showed inverse correlations, one study in pre- and postmenopausal women [84] and the other one only in postmenopausal cases [85]. Breast cancer risk was much lower in postmenopausal women with elevated adiponectin levels in a large prospective case-control study in two North American cohorts [86]. For endometrial cancer, two case-control studies have reported significant inverse correlations which seem stronger in younger women [87] while a prospective case-control study, nested within the EPIC, found a lower risk for women in the highest versus lowest quartiles of adiponectin after adjustment for BMI [88]. The Health Professionals Follow-up Study reported that low adiponectin blood levels was a risk factor for colorectal cancer in men [89], but this finding has not been confirmed in other prospective studies. There are no clear-cut results available for other cancers such as pancreas adenocarcinoma or prostate cancer in nonsmoker populations. Several studies have suggested that the activation of peroxisome proliferator-activated receptor-*γ* (PPAR-*γ*) may be involved in cancer development in obesity, since it inhibits cell proliferation and induces apoptosis in vitro, possibly by antagonizing the activities of NF-*κ*B [90]. 

Obesity has several features for altered PPAR-*γ* function, which include an increased production of PAI-1, and there are diverse studies which show that PAI-1 promotes tumour growth through the inhibition of apoptosis. It is also an indicator of poor prognosis for several cancers such as breast cancer [91]. PAI-1 also seems to be involved in angiogenesis, enhanced cell adhesion, and increased migration [91]. Furthermore, in breast cancer, PAI-1 is implicated in inflammatory response, neutrophil recruitment, and proliferation of smooth muscle cells. PPAR-*γ* dysregulation and PAI-1 overproduction in adipose tissue of obese women may change the breast microenvironment and facilitate local cancer development and/or metastasis [91]. Maury et al. have identified other adipokines that are oversecreted by omental adipose tissue (AT) in obesity and which positively correlate with BMI, insulin resistance, and hypoadiponectinemia [92]. In particular, the circulating concentrations of adipokines such as growth-related oncogene factor *α* (GRO-*α*), tissue inhibitor of metalloproteinases-1 (TIMP-1), and thrombopoietin (TPO) are increased in severely obese women [92, 93]. Therefore, high levels of these molecules are suspected to promote cancerogenesis especially as GRO-*α* is involved in inflammation, angiogenesis, and tumorigenesis [94], and high levels of GRO-*α* and TIMP-1 may play a role by enhancing cell proliferation, antiapoptotic activity, and/or angiogenesis [94], while TPO stimulates the proliferation and differentiation of megakaryocytes [95]. 

### 5.7. Chronic Inflammation and Nuclear Factor-*κ*B System

Obesity is correlated to chronic inflammatory response, with abnormally high cytokine production and the activation of proinflammatory signalling pathways. The subclinical low-grade chronic inflammatory state in obesity might be important in the initiation and promotion of cancer cells [2]. Prospective studies have reported that an increase in circulating C-reactive protein (CRP) is associated with increased risk of colorectal cancer, and, in particular, it has been shown that genetic variation in the CRP gene influences risk of both colon and rectal cancer developments [96]. A case-control study has also provided epidemiological evidence that endometrial carcinogenesis may be promoted by an inflammatory milieu [97]. Of the two different pathways which link inflammation with tumorigenesis, one is an extrinsic pathway mediated by chronic inflammation (inflammation-induced cancer) and the other an intrinsic pathway where genetic alterations, without underlying inflammation, originate a tumor-driven host immune response creating a microenvironment made up of inflammatory cells (cancer-induced inflammation) [98]. Both intrinsic and extrinsic pathways link inflammation and cancer by a number of oncogenes (ras/raf, ReIB, nuclear oncogenes, oncosuppressors, etc.) [98, 99]. Interestingly, abundant literature suggests that energy accumulation plays an important part in the origin of chronic inflammation [100], and, in fact, caloric restriction induces low inflammation. Recent evidence gives support to the idea that the inflammatory response is to increase energy expenditure through feedback to compensate for the excess of energy in obesity, and if this feedback system is not efficient (inflammation resistance), energy expenditure is lowered and energy is conserved which then leads to obesity [100]. 

### 5.8. Crosstalk between Tumour Cells and Surrounding Adipocytes and Migrating Adipose Stromal Cells

Tumour progression is now seen as resulting from the crosstalk between tumour cells and the “normal” cells which surround them. In breast cancer, mature adipocytes are part of the breast cancer tissue and thus contribute to cancer progression as highly specialized endocrine cells capable of modifying breast cancer cell behaviour. It has been proposed that evolving bidirectional crosstalk is functional between breast cancer cells and tumour-surrounding adipocytes, and that the tumour-modified adipocytes are actively involved in tumour progression [101]. Tumour vasculogenesis is also essential in cancer development, and a possible source of progenitor cells for new vessels could be mesenchymal stromal cells (MSCs) which differentiate into different phenotypes, and which, though at low plasma concentrations, can be attracted by pathological signals such as hypoxia or inflammation to specific tumour sites where they promote angiogenesis, driving tumour progression, and become tumour stromal cells. Other than from bone marrow, MSCs may arise from white adipose tissue (WAT) and other sources [102]. It has been suggested that a higher frequency of MSCs in peripheral blood may well be a diagnostic marker for colorectal cancer [103], and changes in circulating MSCs dependent on BMI, possibly mobilized from white adipose tissue, may be addressed to tumors, representing a link between obesity and cancer progression. 

### 5.9. Adipose Tissue and Vascular Growth Factors

Not only do obese patients with different types of cancers have a worse prognosis but also they have an increased risk of metastatic disease and a shorter remission period after treatment, perhaps because angiogenesis may be exacerbated in obesity [104]. In angiogenesis, there is an equilibrium between angiogenic factors, which stimulate the proliferation and migration of endothelial cells, and antiangiogenic factors, which inhibit these activities. Endothelial cells abound in adipose tissue, and they secrete angiogenic factors, such as VEGF and HGF, which have autocrine/paracrine actions and endocrine effects elsewhere. Antiangiogenic factors such as angiostatin, endostatin, and adiponectin are also expressed in adipose tissue [82]. An altered balance between vascular growth factors and inhibitors is typical of obesity, with expansion of the capillary bed in regional fat depots [105], thus possibly contributing to the increased risk of metastatic disease in obese subjects with cancer.

### 5.10. Obesity-Induced Hypoxia

Oxygen levels in the WAT of obese mice are considerably lower than those of lean mice [106], and adipose tissue hypoxia may contribute to cancer risk. In particular, transcription factor HIF-1*α* is correlated with increased metastatic spread and poor prognosis [107]. Studies from Bedogni et al. have previously demonstrated the importance of mild hypoxia of the skin in melanocyte transformation and the development of melanoma [108].

### 5.11. Oxidative Stress

Antioxidant activities may be independently decreased by obesity, thus increasing systemic oxidative stress, in part through the inflammatory process and in part independently of other mechanisms [109]. Increased oxidative damage to DNA may heighten cancer risk.

### 5.12. Shared Genetic Susceptibility

Genetic predisposition to obesity may predispose to the development of certain tumours, and it is now possible to identify in associated regions likely mutual candidate genes for cancer and obesity, independently of diabetes. Recently, a study in Scotland [110] reported that variants of common single nucleotide polymorphism around the melanocortin 4 receptor (MC4R) gene are associated with both obesity and colorectal cancer.

### 5.13. Endocrine Disruptors

Exposure to endocrine disruptors such as stress and food contaminants, which have an effect on the regular activity of estrogens, androgens, and insulin may also affect the simultaneous development of obesity and cancer [111]. 

### 5.14. Energy Homeostasis and Cancer

Coupled to insulin signalling, nutrient sensing pathways have been hypothesized to play a role in carcinogenesis. Several studies are currently examining targets such as AMP-activated protein kinase (AMPK), mammalian target of rapamycin (mTOR), fatty acid synthase, deacetylase SIRT1 (sirtuin 1), and epigenetic modulators [1].

### 5.15. Obesity, Immune Function and Risk of Cancer

Immune system alterations may be important in the higher incidence rates of cancer in obese patients since obesity has been correlated to a reduced immunocompetence in humans. Moulin et al. have recently shown that severely obese patients have a significantly lower natural killer (NK) cell cytotoxic activity as compared to normal individuals matched for age and gender, with lower defence against invaders such as precancerous and cancerous cells [112]. Interestingly, weight loss (26% less of initial weight) is responsible for an increase in NK cytotoxic activity after 6 months from gastric bypass surgery [112]. Lynch et al. have recently shown that omental invariant NKT (iNKT) cell frequencies were lower in patients with severe obesity as compared to lean healthy subjects, thus suggesting a novel role for the omentum in immune regulation and tumor immunity [113].

The following list shows the possible factors involved in the development of cancer in obesity. 

Anthropometric parameters:

 body mass index (BMI) [3],
 weight increase [26, 27],
 visceral fat [18–23].

Lifestyle factors:

 hypercaloric diet [33, 100],
 high intake of animal fat, trans-fatty acids, and refined carbohydrates and low intake of fiber, vegetables, fruit, whole grain carbohydrates, and 25-hydroxyvitamin D (25(OH)D) [34, 37, 38],
 sedentary lifestyle [39, 40].

Biological mechanisms:

 hyperinsulinemia and insulin resistance [35, 41–45, 51, 52],
 IGF-I, IGF-II, and IGF binding proteins [35],
 sex hormones and sex hormone binding globulin [60–67],
 chronic whole body low-grade inflammation [96–100],
 adipose tissue inflammation [70],
 adipose tissue adipokine production (leptin, adiponectin, resistin, PAI-1, growth-related oncogenes, etc.) [71–78, 80–91, 94, 95],
 adipose tissue vascular growth factors [82, 104],
 shared genetic susceptibility [110],
 oxidative stress [109],
 endocrine disruptors [111],
 immune function (NK cells and invariant NKT cells) [112, 113].

## 6. Conclusions

Obesity is responsible for approximately 20% of all malignancies, although its influence is gender and site specific. The association between obesity and a higher cancer risk is mainly due to anthropometric parameters and lifestyle factors which activate different biological mechanisms. Anthropometric parameters are BMI, weight increase, and the amount of body fat, particularly visceral fat. Lifestyle factors include sedentary habits and diet parameters, such as a hypercaloric and/or low-quality diet. The most important biological mechanisms mediating the unfavourable influence of the above factors are hyperinsulinemia and insulin resistance, the activities of IGFs and IGF binding proteins, sex hormones and SHBG, general and adipose tissue low-grade inflammation, changes in adipose tissue production of adipokines and vascular growth factors, oxidative stress, endocrine disruptors, and alterations in immune function. We do not yet have a clear scientific demonstration that avoiding increasing or reducing body weight significantly reduces the risk of cancer; however, the data from the surgical treatment of obese patients with a BMI higher than 40.0 are leading in this direction.
